# Decoding dynamic Ca^2+^ signaling in the vascular endothelium

**DOI:** 10.3389/fphys.2014.00447

**Published:** 2014-11-17

**Authors:** Mark S. Taylor, Michael Francis

**Affiliations:** ^1^Department of Physiology, University of South Alabama College of MedicineMobile, AL, USA; ^2^Department of Pharmacology, University of South Alabama College of MedicineMobile, AL, USA

**Keywords:** Calcium dynamics, endothelium, spatiotemporal signaling, acquisition and analysis, vasodilation

## Abstract

Although acute and chronic vasoregulation is inherently driven by endothelial Ca^2+^, control and targeting of Ca^2+^-dependent signals are poorly understood. Recent studies have revealed localized and dynamic endothelial Ca^2+^ events comprising an intricate signaling network along the vascular intima. Discrete Ca^2+^ transients emerging from both internal stores and plasmalemmal cation channels couple to specific membrane K^+^ channels, promoting endothelial hyperpolarization and vasodilation. The spatiotemporal tuning of these signals, rather than global Ca^2+^ elevation, appear to direct endothelial functions under physiologic conditions. In fact, altered patterns of dynamic Ca^2+^ signaling may underlie essential endothelial dysfunction in a variety of cardiovascular diseases. Advances in imaging approaches and analyses in recent years have allowed for detailed detection, quantification, and evaluation of Ca^2+^ dynamics in intact endothelium. Here, we discuss recent insights into these signals, including their sources of origination and their functional encoding. We also address key aspects of data acquisition and interpretation, including broad applications of automated high-content analysis.

## Introduction

The endothelium plays a pivotal role in vascular function and cardiovascular homeostasis, including regulation of vascular tone, permeability, inflammation, and angiogenesis (Furchgott and Zawadzki, 1980; Vandenbroucke et al., 2008; Davis et al., 2011; Xiao et al., 2013). Endothelial function is inherently linked to cardiovascular health, and endothelial dysfunction is a hallmark of cardiovascular disease (Quyyumi, 2003; Munaron and Pla, 2009). Free intracellular Ca^2+^ concentration directs a wide range of endothelial cell responses, but our understanding of dynamic targeting and titration of Ca^2+^ signals within the intact endothelium remains surprisingly rudimentary. New findings have exposed a complex mosaic of physiologic endothelial Ca^2+^ signals (Duza and Sarelius, 2004; Kansui et al., 2008; Ledoux et al., 2008). These spatially and temporally discrete events comprise a highly structured language along the vascular intima, allowing for selectivity and coordination of cellular responses. Here we discuss recent insights into Ca^2+^ dynamics and implications for Ca^2+^-effector coupling in the endothelium. We also address advances in signal tracking and quantification that will play a crucial role in the elucidation of endothelial Ca^2+^ signaling patterns and the development of new physiologic models.

## Ca^2+^ dependent signaling in the endothelium

Endothelial Ca^2+^ targets include a variety of cell effectors. Some contain intrinsic Ca^2+^-binding motifs such as C2 domains. However, most are dependent on calmodulin (CaM), a Ca^2+^-binding protein containing a high-affinity EF-hands motif that allows for cellular responses with nanomolar Ca^2+^ changes. Key Ca^2+^-CaM dependent endothelial effectors include nitric oxide synthase (eNOS) that produces the diffusible vasorelaxing factor nitric oxide (NO) (Ignarro et al., 1987; Busse and Mulsch, 1990), as well as small/intermediate conductance Ca^2+^-activated K^+^ channels (K_Ca_), K_Ca_2.3, and K_Ca_3.1 (Xia et al., 1998; Crane et al., 2003; Dora et al., 2008) that elicit vasorelaxation through endothelium derived hyperpolarization (EDH) of medial vascular smooth muscle (Murphy and Brayden, 1995; Chaytor et al., 1998; Emerson and Segal, 2000; Félétou and Vanhoutte, 2000; Burnham et al., 2002; Bychkov et al., 2002; Taylor et al., 2003). Endothelial Ca^2+^ rise also contributes to phospholipase A2-mediated production of arachidonic acid metabolites, including the vasorelaxing factors prostacyclin and epoxyeicosatrienoic acids (Jaffe et al., 1987; Campbell and Fleming, 2010). The predominant factors vary considerably among vascular beds. NO exerts a major influence in large vessels whereas hyperpolarizing mechanisms predominate in small vessels and the microcirculation (de Wit and Wölfle, 2007). The primary functional consequence of endothelial Ca^2+^-effector recruitment is vasodilation. However, vasoconstricting factors such as endothelin may also be released as a result of Ca^2+^ elevation (Marsen et al., 1996), particularly under conditions of injury or disease. So, how does a seemingly ubiquitous signal, Ca^2+^, selectively recruit different endothelial effectors with diverse functional roles? A closer look at the intact endothelium has revealed a fundamental signaling paradigm involving repetitive spatially restricted Ca^2+^ transients. As discussed below, these signals are capable of exerting effector-specific influences on vascular function and likely contribute to diverse profiles of endothelial response.

### Endothelial Ca^2+^ dynamics

Over the past few decades, most Ca^2+^ measurements have involved assessments of whole-field epifluorescence at rates slower that 1 Hz, and often at supra-physiological levels of cell/tissue stimulation. While this approach is useful for tracking global trends in Ca^2+^ over protracted time scales and evokes acute fluorescence signals large enough to quantify unequivocally, it does little to elucidate the spatial and temporal detail of Ca^2+^ dynamics. Insights into physiologic Ca^2+^ signaling have come largely from the use of single-excitation fluorescent Ca^2+^ probes in high-speed confocal imaging applications, particularly within intact tissue preparations. Evaluations of isolated arterial segments have revealed a plethora of spatially and temporally discrete Ca^2+^ signals. In arterial smooth muscle, spontaneous localized Ca^2+^ transients (e.g., Ca^2+^ sparks and sparklets) as well as asynchronous and synchronous Ca^2+^ waves control vascular tone through coordination of cellular activation and feedback regulation of constriction (Nelson et al., 1995; Santana et al., 2008; Mufti et al., 2010). Hints of similar dynamic signals in the endothelium were observed as Ca^2+^ waves coursing through isolated cells (Neylon and Irvine, 1990; Isshiki et al., 2004). Unfortunately, detailed imaging of endothelial Ca^2+^ activity *in situ* has been quite challenging because of the general inaccessibility of the vascular intima (i.e., on the internal surface and only one-cell thick). Various strategies have been employed for vascular endothelial imaging including intravital microscopy (Bagher et al., 2011), myograph-mounted arterial segments (Schuster et al., 2001), exposed endothelial tubes (Socha et al., 2012), and pinned-open artery segments (Marie and Bény, 2002). Open-artery preparations have proven quite useful. This involves cutting artery segments open longitudinally and pinning them to silicone blocks, thereby making the endothelial layer accessible to rapid indicator loading and *en face* confocal imaging (see Ledoux et al., 2008). In such preparations, many cells (~ 200 with 20X objective) can be evaluated in a single plane while preserving the native environment, including lamina attachments and cell–cell communication. Cell-permeant, single-excitation fluorescent dyes like Fluo-4 AM have enabled rapid scanning. Implementation of the transgenic GCaMP2 mouse model has also proven beneficial by providing an endothelial-expressed Ca^2+^-dependent fluorophore that avoids spill-over smooth muscle fluorescence and improves signal detection and quantification (Kotlikoff, 2007; Ledoux et al., 2008).

Basal endothelial Ca^2+^ events were first characterized in mouse mesenteric arteries (Ledoux et al., 2008). Termed Ca^2+^ pulsars, these events resemble muscle cell Ca^2+^ sparks although somewhat broader in spatial range and duration. Unlike Ca^2+^ sparks that emit from ryanodine receptors (RyR), Ca^2+^ pulsars release intermittently from the endoplasmic reticulum through clusters of inositol 1,4,5-trisphosphate receptors (IP_3_Rs). Pulsars are similar to Ca^2+^ puffs, localized Ca^2+^ events previously described in Xenopus oocytes (Parker et al., 1996). Liberated from distinct IP_3_R clusters, Ca^2+^ puffs increase in frequency with increasing IP_3_, ultimately expanding into cell-wide waves. This transition to propagating waves occurs through IP_3_ sensitization of neighboring IP_3_R clusters, leading to a chain reaction of Ca^2+^-induced Ca^2+^ release (Foskett et al., 2007). Ca^2+^ pulsars occur basally in mesenteric arteries under resting conditions (at 37°C and no flow), and these ongoing events are blocked by inhibiting phospholipase C (Ledoux et al., 2008), the enzyme that produces IP_3_. Stimulation of the mesenteric artery endothelium with acetylcholine (ACh) increases the number of Ca^2+^-emitting sites along the intima and augments the frequency of events occurring at previously active sites. Thus, Ca^2+^ pulsars can be tuned acutely by G_*q*_ protein-coupled receptor (G_*q*_PCR) stimulation.

Ca^2+^ pulsar events occur predominantly around the nucleus and at distinct myoendothelial junction (MEJ) sites where endothelial cell projections form close contacts (and often heterocellular gap junctions) with smooth muscle cells through holes in the internal elastic lamina (Sandow et al., 2002, 2009; Ledoux et al., 2008). These sites correspond with densities of IP_3_Rs. The primary functional target of pulsars appears to be K_Ca_ channels, particularly K_Ca_3.1 channels that are highly concentrated in the plasma membrane of myoendothelial projections. Importantly, this ongoing Ca^2+^-effector coupling exerts a persistent EDH influence (Ledoux et al., 2008) capable of relaxing underlying vascular smooth muscle and modulating arterial tone. The vascular smooth muscle may itself directly influence endothelial Ca^2+^ signals (Yashiro and Duling, 2000). In particular, smooth muscle IP_3_ generated by G_*q*_PCR stimulation (i.e., via sympathetic activity and circulating hormones) may be communicated across MEJs, augmenting endothelial Ca^2+^ dynamics (Lamboley et al., 2005). Indeed, addition of the α_1_-adrenergic receptor agonist phenylephrine increases endothelial Ca^2+^ events in mesenteric arteries (Kansui et al., 2008) and recruits new axially propagating Ca^2+^ wavelets in previously inactive endothelial cells of skeletal muscle feed arteries (Tran et al., 2012). This communication may allow endothelial influences to be adjusted relative to vasoconstrictor stimulation, providing real-time feedback regulation of vascular tone.

Additional players have recently been implicated in intrinsic endothelial Ca^2+^ signals, namely the transient receptor potential (TRP) non-selective cation channels (Di and Malik, 2010). In particular, certain vanilloid family channels (TRPV4) have been found to produce localized Ca^2+^ transients along the plasma membranes of mouse mesenteric artery endothelium (Sonkusare et al., 2012). Likely obscured by broader pulsar events, these small, membrane-delimited Ca^2+^ sparklets can be unmasked by depletion of internal stores and treatment with the TRPV4-stimulating compound GSK1016790A. Like Ca^2+^ pulsars, the TRPV4 sparklets couple to nearby K_Ca_ channels. Notably, when ER Ca^2+^ stores are not depleted, TRPV4 stimulation causes widespread whole-cell Ca^2+^ dynamics. Similarly, in the endothelium of rat cerebral arteries, activation of ankyrin-associated TRPA1 channels causes recruitment of discrete Ca^2+^ events that spread as propagating waves (Qian et al., 2013). Together, these findings suggest membrane-delimited TRP channel events may solicit broader internal Ca^2+^ store release events. Indeed, the interplay between external and internal Ca^2+^ sources may contribute to a wide spectrum of conditional Ca^2+^ dynamics and effector recruitment profiles.

### Idiosyncratic Ca^2+^-effector coupling and functional encoding of Ca^2+^ dynamics

Fundamental endothelial Ca^2+^ signals (pulsars and sparklets) primarily target K_Ca_3.1 channels concentrated in densities along the endothelial basolateral membrane and myoendothelial junctions. However, related K_Ca_2.3 channels are distributed quite differently, residing primarily along endothelial cell–cell borders, associated with the plasma membrane protein caveolin (Sandow et al., 2006; Absi et al., 2007). Notably, certain TRP channels (e.g., TRPV4 and TRPA1) distribute preferentially with K_Ca_3.1 or K_Ca_2.3 channels (Earley et al., 2009; Ma et al., 2013), perhaps due to conditional TRP association with caveolin (Rath et al., 2009). Overall, this suggests differential Ca^2+^ signal targeting of K_Ca_ isoforms. Recent findings suggest that in addition to direct EDH signaling, K_Ca_/TRP coupling may directly influence the endothelial Ca^2+^ signals themselves. Specifically, Ca^2+^-dependent hyperpolarization may increase the driving force for further Ca^2+^ influx through TRP channels, allowing positive feedback augmentation of the original Ca^2+^ signal. In support of this scenario, ACh-induced endothelial Ca^2+^ dynamics are substantially higher in normal mesenteric arteries compared to those from mice lacking K_Ca_3.1 and K_Ca_2.3 channels, and this Ca^2+^-facilitating influence of K_Ca_ channels is blocked by inhibition of TRPV4 activity (Qian et al., 2014). Taken together, these findings imply that the specific arrangement of specific ion channels within endothelial cells is a key determinant of the prevailing Ca^2+^ signals and effector recruitment profiles.

Endothelial NOS resides in two functional pools, one associated with caveolin in the cell periphery, and the other in the membrane of the Golgi apparatus (Liu et al., 1997; Andries et al., 1998; Rath et al., 2009). The provisional association of K_Ca_2.3, TRPV4, and eNOS with caveolin suggests their possible interaction. Indeed, SK3 overexpression increases the NO contribution to ACh-induced vasodilation (Brähler et al., 2009), and relaxation of rat pulmonary arteries via TRPV4 activation is linked to both NO and K_Ca_ channel activity (Sukumaran et al., 2013). Whether such scenarios involve targeting of plasma membrane eNOS, by K_Ca_2.3-enhanced TRPV4 Ca^2+^ signals, remains to be determined.

Expansion or redirection of inherent Ca^2+^ signals is crucial to endothelial function. Stimuli including GPCR agonists and TRP channel activators increase the occurrence of endothelial events, including recruitment of new active sites and increased firing frequency (Ledoux et al., 2008; Qian et al., 2013), and both effects are linked to proportional arterial dilation. The overarching implication is that endothelial vasoregulation is encoded by both binary and analog Ca^2+^ signaling modes. That is to say, discrete sites are either on or off (binary), and once on, the attributes of the events are tunable over some range (analog). In addition to frequency, analog signaling components include magnitude, duration, and spatial spread of Ca^2+^ events, all of which could affect the type and extent of effector recruitment. Ultimately, definitive tracking of discrete Ca^2+^ signaling patterns will be needed to reveal the nature and capacity of parameter expansion and decipher the idiosyncrasies of endothelial function and dysfunction.

### Quantifying and profiling endothelial Ca^2+^ dynamics

Given the inherent complexity of endothelial Ca^2+^ signals, a key challenge moving forward will lie in our ability to adequately and comprehensively characterize Ca^2+^ activity along the intact intima. Disparate approaches have been employed to measure and analyze Ca^2+^ data, often applying arbitrary, if any, selection criteria and providing little explication of spatial and temporal parameters. Regardless of experimental preparation and approach, some crucial criteria for acquisition and analysis should be considered. First, spatial or temporal under-sampling of Ca^2+^ fluorescent signals washes out discrete dynamics or misses them altogether. High-speed confocal imaging systems, particularly spinning disk platforms with high-quantum efficiency cameras, offer sub-micron spatial resolution with fast acquisition rates and high signal to noise ratios. Analysis of dynamic Ca^2+^ activity typically involves hand selection of an event within an image time-series and manual placement of a region of interest (ROI, often a small box or circle) around each event site for measurement of average fluorescence. Albeit straight-forward, this approach is tedious, time-consuming and prone to user-bias and error. Recent efforts have produced automated detection and analysis algorithms to extract signals from continuous image sequences. Evidence suggests that in addition to saving time and resources, automated analyses can avoid inconsistencies of manual analysis and identify signaling signatures within complex fluorescence data. Multiple software applications, particularly in neurobiology, have employed independent component analysis and watershed image segmentation to define individual cells within dense fields and to track region-specific deflections of Ca^2+^-dependent fluorescence (Mukamel et al., 2009; Wong et al., 2010; Watters et al., 2014). Separate automated analysis software has been applied to discern Ca^2+^ spiking and oscillation patterns in various cell types, including plant epidermal cells (Russo et al., 2013), cardiac myocytes (Janicek et al., 2013), and T-cells (Salles et al., 2013). The algorithm LC_Pro was recently developed to track the diverse Ca^2+^ events in the vascular endothelium (Francis et al., 2012, 2014). Incorporated as a plug-in with ImageJ freeware, this statistically rigorous program distinguishes dynamic fluorescence signals from background noise, and follows the spatial profile of each Ca^2+^ event with time. It automatically assigns ROIs to event spatial centers and returns output quantifying relevant field and event parameters (e.g., sites, events, amplitude, duration, and spatial spread). The algorithm also allows “batch” analysis of multiple parallel data sets. Such analysis is particularly useful because it can generate complete parameter distributions and provide practical quantification of replicate data sets or complex signal changes following perturbation (i.e., endothelial stimulation). For instance, relative changes in binary (i.e., sites) and analog signals (i.e., amplitude, duration, spread, single-site frequency) can be automatically calculated, plotted, and statistically evaluated in a series of experiments without intermediate data processing by the user. Regardless of specific approach, stringent analysis of large data sets will be a necessary step in decoding Ca^2+^ dynamics.

Overall, automated analysis approaches have become useful for defining cell boarders, discerning cellular/subcellular fluorescence signals from statistical noise, and providing comprehensive quantitation of component signal parameters. Current limitations of such approaches primarily stem from narrowly targeted applications and disparate processing algorithms that can contribute to false-negatives or false-positives when data fall outside an optimal range. As discussed below, extended initiatives should promote more widely applicable tools capable of reducing complex and heterogeneous data sets to intuitive indices of functional signaling.

## Future directions and challenges

Looking forward, careful consideration should be given to the limits and liabilities of experimental approaches. While high-speed confocal imaging is valuable for signal resolution, it imposes certain experimental restrictions, including thin-plane sampling (~ 1 μm) and low tolerance for tissue movement. Wire-mounted or pressurized arteries can be studied as intact segments, but endothelial exposure is very limited. On the other hand, open vessel preparations expose vast endothelial fields in a single plane but sacrifice tubular structure and functional assessment. Imposing rigid acquisition criteria may be impractical. Rather, strategies should be implemented and optimized to ensure adequate spatial and temporal resolution and prevent image artifact. Importantly, Nyquist sampling criteria should be satisfied (i.e., sampling time and spatial intervals ≤½ of smallest signal duration and size) to ensure reliable signal quantification without signal aliasing or distortion. Automated detection software such as LC_Pro may be useful for optimization by identifying which spatial and temporal acquisition conditions achieve convergence of parameter values while avoiding oversampling. Stack registration software can also be employed as a data processing step to correct for spatial drift (x-y movement) (Thévenaz et al., 1998). Fast piezo focus for rapid z-axis stacking is very useful not only for acquiring depth information within the sample but also for compensating for z-axis drift. It should be noted that inclusion of z-stacks as well as increased exposure times and pixel-binning can all improve certain aspects of image quality but may lead to a loss of overall spatiotemporal resolution, and should be employed with caution.

While ROIs are convenient for assessing spatially discrete Ca^2+^ dynamics, these fixed sampling windows can be problematic when tracking widely disparate signals. For instance, a focal event occupying only a small fraction of an ROI will yield a very small average fluorescence change (amplitude) compared to a broad wave passing through the same ROI, even if both have the same absolute signal intensity. Also, a single fixed ROI may detect spill-over signal from nearby events over time, distorting quantification of site-specific activity. In addition, fixed ROI sampling can promote artifact due to x-y drift by allowing hot spots or even regions of high or low background fluorescence to move into and out of the measured region (i.e., box) over time. Such issues may be resolved by tracking each event individually in space and time, allowing a signal to define its own transient polygonal ROI without establishing a permanent sampling window. Finally, dynamic Ca^2+^ events are often represented as ratios of relative fluorescence change within an ROI (i.e., F/F_0_, where F_0_ is a user-defined base fluorescence value). Defining appropriate base values can be challenging, particularly when photobleaching causes signal drift or high dynamic activity obscures the background. In addition to background correction algorithms, linear regression of time-course data can be applied for F_0_ designations (Francis et al., 2012). Caution is warranted when expressing data as ratios since very low or very high base values can dramatically inflate or deflate F/F_0_ values.

## Perspectives

Because Ca^2+^ dynamics are complex, data are typically represented by a profile of parameters or parameter distributions rather than a single scalar value. This multidimensional description has the capacity to distinguish Ca^2+^ signaling modalities, such as responses to distinct stimuli or among different vascular beds. Notably, because perturbations can increase some parameters and decrease others, quantification, comparison, and interpretation of data can be quite complex. Future analysis and meta-analysis approaches will need to address this complexity, perhaps by tracking trends in global distribution profiles or by defining cumulative metrics that combine parameters into standard indices. Additional indices might also include site distribution, cell heterogeneity, and event synchrony. A growing number of analysis algorithms are available as open source packages and plug-ins, making them not only widely accessible but amenable to customization. The hope is that eventually, a suite of analysis modules could be employed universally for parameter compilation, data mining, and pattern recognition. This would allow a standard analysis scheme for comparison of data sets across labs and preparations. Still, the onus ultimately falls on investigators to extract data or composite parameters germane to their specific experimental questions.

## Conclusions

New insights suggest the endothelium functions as a continuum of dynamically regulated influences that are always engaged and are constantly adjusted. The prevailing Ca^2+^ signaling modalities and effector distributions likely underlie the distinct functions of different circulations. Further dissection of this diverse activity will allow for identification of sub-modalities, and potentially distinct cell phenotypes within the intima. We submit that shifts in prevailing Ca^2+^ dynamics necessarily impact blood pressure and flow and may predict disease. Indeed, endothelial dysfunction is an overarching feature of cardiovascular pathology. It is therefore particularly imperative that future studies shift away from assumptions based on global Ca^2+^ changes and broad cellular protein concentrations and focus on spatially and temporally relevant aspects of real-time signaling. Ultimately, the development of a definitive and predictive model of endothelial function should allow for elucidation of specific control points and therapeutic targets.

### Conflict of interest statement

The authors declare that the research was conducted in the absence of any commercial or financial relationships that could be construed as a potential conflict of interest.
